# Watershed Sediment Losses to Lakes Accelerating Despite Agricultural Soil Conservation Efforts

**DOI:** 10.1371/journal.pone.0053554

**Published:** 2013-01-09

**Authors:** Adam J. Heathcote, Christopher T. Filstrup, John A. Downing

**Affiliations:** Department of Ecology, Evolution, and Organismal Biology, Iowa State University, Ames, Iowa, United States of America; Lakehead University, Canada

## Abstract

Agricultural soil loss and deposition in aquatic ecosystems is a problem that impairs water quality worldwide and is costly to agriculture and food supplies. In the US, for example, billions of dollars have subsidized soil and water conservation practices in agricultural landscapes over the past decades. We used paleolimnological methods to reconstruct trends in sedimentation related to human-induced landscape change in 32 lakes in the intensively agricultural region of the Midwestern United States. Despite erosion control efforts, we found accelerating increases in sediment deposition from erosion; median erosion loss since 1800 has been 15.4 tons ha^−1^. Sediment deposition from erosion increased >6-fold, from 149 g m^−2^ yr^−1^ in 1850 to 986 g m^−2^ yr^−1^ by 2010. Average time to accumulate one mm of sediment decreased from 631 days before European settlement (ca. 1850) to 59 days mm^−1^ at present. Most of this sediment was deposited in the last 50 years and is related to agricultural intensification rather than land clearance or predominance of agricultural lands. In the face of these intensive agricultural practices, traditional soil conservation programs have not decelerated downstream losses. Despite large erosion control subsidies, erosion and declining water quality continue, thus new approaches are needed to mitigate erosion and water degradation.

## Introduction

Soil erosion and nutrient loss from agricultural and urban lands are important problems facing inland, coastal, and marine waters [1]. Despite the US spending $5 billion annually to limit soil and nutrient losses from fields [2], intensive agricultural practices still threaten clean water resources [3].

**Figure 1:**
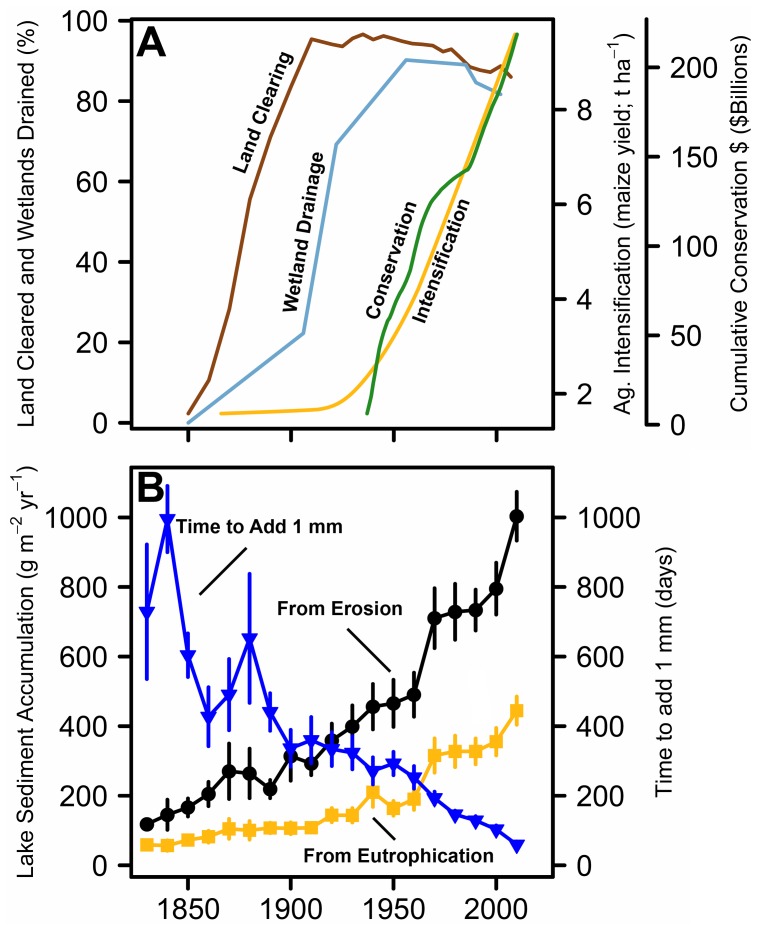
Changing agricultural practices and regional lake sedimentation rates since European settlement, shown as decadal averages across all 32 lakes. (A) Percent land in farms (brown), percent of wetlands drained (light blue), maize yield (t ha^−1^) (yellow), and cumulative USDA financial assistance (inflation adjusted) for soil and water conservation programs in the USA (green). (B) Average regional lake mass accumulation rates for erosional (black) and in-lake (fueled by nutrient enrichment; yellow) derived sediment (g m^−2^ yr^−1^). The time for lakes to add one mm of sediment is also shown (dark blue; days). Error bars represent ±1 standard error. Agricultural data were summarized from the United States Department of Agriculture’s Census of Agriculture (1850–2007) and the National Agricultural Statistics Service. Annual maize yield data were fitted to a LOWESS model.

Historical agricultural development in the Midwestern United States primarily consisted of large-scale landscape change and agricultural intensification. Historical change occurred in three phases (Fig. 1A) that are consistent across the agricultural regions of North America. (I) Land clearing of the native wet tallgrass prairie vegetation for agriculture began around 1850 and was 95% complete by 1910. (II) Land drainage by stream channelization and subsurface drains began around 1850, draining 70% of wetlands by 1920 [4]. (III) 1950s intensification of agriculture via increased average farm size, mechanization, fertilizer, and biocide application increased yields almost 4-fold [5].

Soil erosion mitigation programs began in 1935 and the US federal government has spent $294 billion (inflation adjusted) since then to support these programs nationwide (Figure 1A). Programs commonly paid farmers to plant cover crops, but retiring farmlands to native vegetation has been more common since 1985 [6]. According to the U.S. Department of Agriculture National Resource Conservation Service (USDA NRCS; http://www.ia.nrcs.usda.gov), in the state of Iowa alone, $53 million was spent on conservation programs, almost half of which went to installing practices such as grassed waterways, terraces, and other sediment and nutrient management strategies. The success of these programs in reducing sedimentation in some regions has been demonstrated [7], but other studies have questioned whether current soil conservation methods are enough to curb these problems, especially in areas where agricultural land use is most intense [8]. It has been suggested that the heart of this controversy is based on the prevalent application of generalized models to estimate soil loss changes, in lieu of actual measurements. There has been a call for more direct measurements of erosion in order to better inform models and more effectively implement conservation practices [9].

We measured the flux of erosional sediment to lakes over the last 180 years and quantified in-lake production of organic matter caused by accompanying nutrient runoff. The lakes in this study are located in an intensively agricultural region where erosion and nutrient mitigation efforts have been intense. Previous studies have used sediment to examine the correlation of agricultural practices with erosion rates with sometimes conflicting results, but these studies have often only considered a small number of lakes [10], [11], [12]. Here we examine a regional lake data set (32 lakes) and show that, on average, sediment accumulation rates have increased exponentially since the intensification of agriculture in the 1950s and that up to 75% of deposited sediment derives from erosion, despite efforts at control. This suggests the need for new approaches to permit the coexistence of productive agriculture and healthy water.

## Materials and Methods

Thirty-two glacially formed (ca. 10,000–13,000 years old) lakes in the intensively agricultural Midwestern state of Iowa, USA, were selected to characterize the history of eroded soil transport and sediment delivery in the region (Table 1). Estimates from 2002 land cover data indicate that some form of conservation practices was present in the watersheds of all lakes (average: 6% of area) and agriculture was the dominant land use (average: 50% of area) (Iowa Geological and Water Survey, http://www.igsb.uiowa.edu). A sediment core was collected from a representative location in each lake, near the average depth, and sediment mass accumulation rates (MARs) were calculated based on ^210^Pb dated core intervals [13]. Two lakes (Black Hawk Lake and Storm Lake) had an additional core taken from a secondary basin. Loss-on-ignition analysis [14] was performed on all dated sections to estimate the proportion of sediment derived from erosional inputs versus in-lake processes. Lake locations and basic water quality information are presented in Table S1. All lakes sampled in this study were publically owned and managed by the State of Iowa. No specific permits were required for the described field studies. Permissions to collect sediment cores from public lakes in this study were given by the Iowa Department of Natural Resources.

**Table 1:** Lake and watershed sizes, maximum ^210^Pb ages at the bottom of cores (± SE), historic and modern sediment total (erosional+in-lake) mass accumulation rates (MAR; ±SE) for the sediment cores taken from 32 lakes in this study.

Lake Name	Lake Area (km^2^)	Watershed Area (km^2^)	Max ^210^Pb Date(± Years)	Historic MAR(g cm^−2^ yr^−1^)	Modern MAR(g cm^−2^ yr^−1^)
Black Hawk Lake	3.1	53.2	1895±20	0.065±0.036	0.120±0.006
Black Hawk Lake (Slough)	0.7	53.2	1881±64	0.081±0.140	0.418±0.034
Burt Lake	0.8	22.4	1829±37	0.033±0.033	0.338±0.020
Center Lake	0.9	1.9	1835±31	0.055±0.052	0.130±0.006
Clear Lake	14.9	38.8	1929±38	0.005±0.004	0.005±0.001
Crystal Lake	1.0	7.4	1857±22	0.053±0.035	0.256±0.014
Diamond	0.6	6.6	1831±34	0.019±0.016	0.201±0.012
East Lake Okoboji	7.4	47.5	1799±28	0.035±0.028	0.142±0.007
Five Island Lake	3.9	31.5	1863±36	0.052±0.053	0.230±0.014
High Lake	1.9	6.3	1889±67	0.040±0.076	0.186±0.016
Ingham Lake	1.4	3.7	1813±39	0.010±0.009	0.098±0.005
Iowa Lake	3.2	36.3	1866±35	0.046±0.048	0.218±0.011
Lake Cornelia	1.0	3.0	1755±96	0.006±0.009	0.101±0.004
Lake Minnewashta	0.5	1.2	1825±21	0.044±0.025	0.260±0.011
Little Spirit Lake	2.4	5.8	1820±45	0.027±0.030	0.193±0.008
Little Wall Lake	1.0	0.8	1792±44	0.013±0.016	0.063±0.004
Lost Island Lake	4.7	20.9	1814±33	0.006±0.005	0.088±0.004
Lower Gar Lake	1.0	40.5	1829±23	0.024±0.015	0.184±0.007
Morse Lake	0.4	1.2	1794±62	0.012±0.016	0.173±0.010
North Twin Lake	1.8	8.4	1849±39	0.032±0.035	0.116±0.007
Pickerel Lake	0.7	6.6	1843±41	0.028±0.032	0.160±0.009
Rice Lake	4.0	61.9	1873±9	0.012±0.002	0.037±0.002
Silver Lake (Dickinson Co.)	4.2	60.0	1823±42	0.013±0.012	0.106±0.004
Silver Lake (Palo Alto Co.)	2.6	33.6	1828±29	0.021±0.016	0.207±0.013
Silver Lake (Worth Co.)	1.3	7.0	1863±32	0.004±0.002	0.005±0.001
Storm Lake	12.3	55.7	1929±21	0.038±0.020	0.161±0.014
Storm Lake (Inlet)	0.4	55.7	1836±128	0.0374±0.110	0.449±0.043
Trumbull Lake	4.8	191.5	1894±41	0.134±0.169	0.152±0.011
Tuttle Lake	9.2	496.7	1822±56	0.016±0.021	0.180±0.010
Upper Gar Lake	0.1	0.8	1869±29	0.037±0.030	0.071±0.003
Virgin Lake	0.9	4.3	1838±21	0.017±0.009	0.135±0.007
West Lake Okoboji	15.6	61.0	1813±30	0.029±0.024	0.099±0.003
West Swan Lake	1.5	35.0	1829±22	0.013±0.006	0.095±0.005
West Twin Lake	0.4	0.5	1863±37	0.059±0.066	0.205±0.012

Previous studies in this region have conservatively estimated that 42% of the sediment organic matter is derived from terrestrial sources [4], so this proportion was added to the terrestrially derived inorganic fraction [15] to determine total erosional inputs. The remaining organic matter plus the fraction of calcium carbonate were assumed to be derived from in-lake processes stimulated by nutrient enrichment. Average MARs were calculated based on the average across all lakes for each decade from 1830–2010 (*n* = 459). Very few dated sections were available prior to 1830 (*n* = 8), so sections from 1760–1839 were combined.

Cumulative sediment delivery from the watershed was calculated by estimating average annual erosional deposition to lakes from a fitted locally weighted regression model (LOWESS; span = 0.67) [16] and multiplying by total lake area to estimate total erosional deposition. The total erosional deposition was then divided by total watershed area to determine the cumulative erosional loss to lakes from the watershed per unit area.

## Results and Discussion

Sediment cores, dated with the radio-isotope ^210^Pb, revealed that sediment deposition from erosion and internal production has increased continuously since settlement and that sediment deposition and agricultural changes followed similar chronologies (Figure 1B). Total mass accumulation rates (MARs) of sedimentation in lakes prior to European settlement and at present are given in Table 1. The sediment deposition from erosion increased from 149 g m^−2^ yr^−1^ in 1850 to 986 g m^−2^ yr^−1^ by 2010 and the average deposition rates for internally-produced sediment increased from 58 g m^−2^ yr^−1^ to 434 g m^−2^ yr^−1^. Erosional sediment delivery and in-lake produced deposition both increased by approximately 7-fold since initial settlement of the region. The largest increases in sediment deposition occurred after 1950, concurrent with agricultural intensification. Average time to accumulate one mm of sediment decreased from 631 days before European settlement (ca. 1850) to 59 days mm^−1^ at present (Fig. 1B).

**Figure 2:**
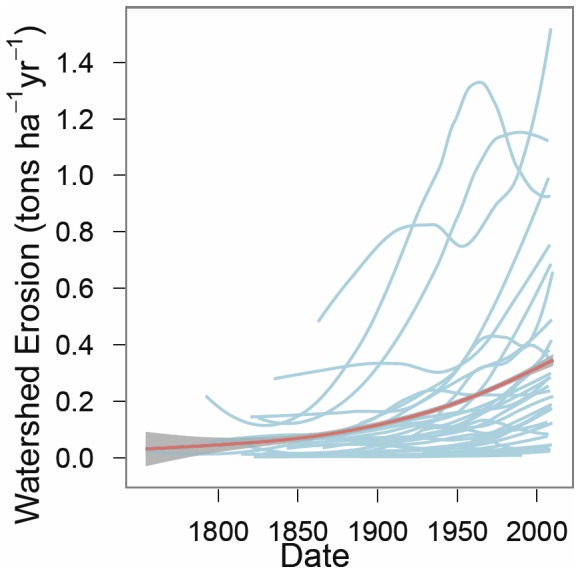
Watershed erosion (tons ha^−1^ yr^−1^) versus time for the 32 lakes in this study since 1800. Red line represents average rate of erosion (LOWESS smoothed fit to the data) from the watershed across all 32 lakes in this study, surrounded by the 95% confidence region. Blue lines represent LOWESS smoothed fits for each of the individual lakes and are included to visualize variability among lakes.

Sediment deposition from erosion represents a median loss of 15.4 t ha^−1^ (mean: 27.6 t ha^–1^) delivered to lakes from the watersheds over the length of time covered in this study (∼180 years) and was the source of up to 75% of total sediment deposited in these lakes. Cumulative erosion calculated from deposition in lakes ranged from 0.8 to 132.8 t ha^−1^. A LOWESS smooth fit to erosion data across all lakes indicates an average increase from 0.1 t ha^−1^ yr^−1^ in 1850 to 0.4 t ha^−1^ yr^−1^ in 2010 (Figure 2). Individual erosion losses from watersheds for each of the 32 lakes are presented in Figure S1.

The eroded soil that leaves watersheds can be a small percentage of total upland erosion in some systems [9]; however, increases in delivery seen in these lakes may reflect increased erosion of topsoil as well as destabilization of upland soil previously deposited as unstable alluvium (e.g., stream bank and bed erosion) [17]. The estimates from this study, therefore, should be considered a measurement of annual net loss of erosional materials from the watersheds rather than a direct measurement of annual topsoil loss.

Eroded sediment and run-off from watersheds with intense agriculture also delivers particulate and dissolved phosphorus (P) [18], which led to increased in-lake production through nutrient enrichment of these systems (Figure 1B). Monitoring data indicate that many of the lakes in this study were eutrophic (Total P>30 µg/l) and highly productive by the mid-1970’s [19] and data from our study show that the largest increases of internally-produced material began around 1950. The efficiency with which herbivores consume additional organic matter decreases with eutrophication because increasingly large and inedible algae may be favored under these conditions [20], [21], which has led to increased deposition of internally-produced material to the sediments. On average, this material accounts for up to 31% of the sediment in these systems and the rate of deposition has nearly tripled since 1950.

Despite well-intentioned efforts, recent changes in farming have increased the potential for erosional deposition of sediment and nutrients. As demand for farm products increased, farms aggregated and expanded onto previously unfarmed slopes and riparian buffer zones [8]. As a result of this consolidation, average farm size in this region increased from 68 ha in 1950 to 134 ha in 2007, despite a 10% decrease in the total area in farmland. Maize yields rose geometrically after 1950 (Figure 1A) due in part to increasing use of chemical fertilizers and intensive row-cropping practices. Both practices have been linked to increasing organic matter transport and burial in lakes and are strongly correlated with agricultural intensification rather than initial land clearance [4].

The complex conveyance processes that transport eroded topsoil out of watersheds, specifically when temporarily stored as colluvium or alluvium, may cause a lag time between changes in land-use (i.e., source erosion) and sediment delivery [22]. Sediment delivery in regions where agricultural influences have not been as intensive in modern years have shown peaks in sediment delivery by 1960 [23] and others have found lake sediments to reflect landscape changes in watersheds within as few as 12 years [11]. The absence of any discernible peak in the rate of sediment delivery across our study sites and the long timescale of our analysis (180 years) suggest that lags in transport of stored sediment alone cannot sufficiently explain the exponential increases in sediment delivery seen in these lakes.

Possibly, erosion and sediment deposition would have been more severe without erosion control subsidies. Soil erosion mitigation programs were created in response to excessive losses of soil from farmers’ fields and water quality concerns. Despite these efforts, however, sediment deposition downstream has not decelerated in one of the most intensively agricultural regions. Our results suggest that decreasing downstream sediment accumulation and water impairment will require more than voluntary programs of subsidized farmland easement. Projected increased global demands for food, energy, and fiber [5] imply that even current erosion rates will be exceeded without better land management practices. Practices aimed at decreasing hydrologic connectivity, surgically stabilizing the most erosion-prone parcels, slowing water transport, restoring riparian corridors, and converting to low intensity agricultural practices may be more efficient options.

## Supporting Information

Figure S1 **Watershed erosion (tons ha^−1^ yr^−1^) versus time for each of the lakes in this study.** Black lines represent a LOWESS smoothed fit to the data.
(TIF)

Table S1 **Location and water quality characteristics for the 32 lakes in this study.**
(DOC)
