# Supplementary figures and images for: Watershed Sediment Losses to Lakes Accelerating Despite Agricultural Soil Conservation Efforts
Source: PLoS One. 2013 Jan 9;8(1):e53554. doi: 10.1371/journal.pone.0053554 (PMC3541183; doi:10.1371/journal.pone.0053554)

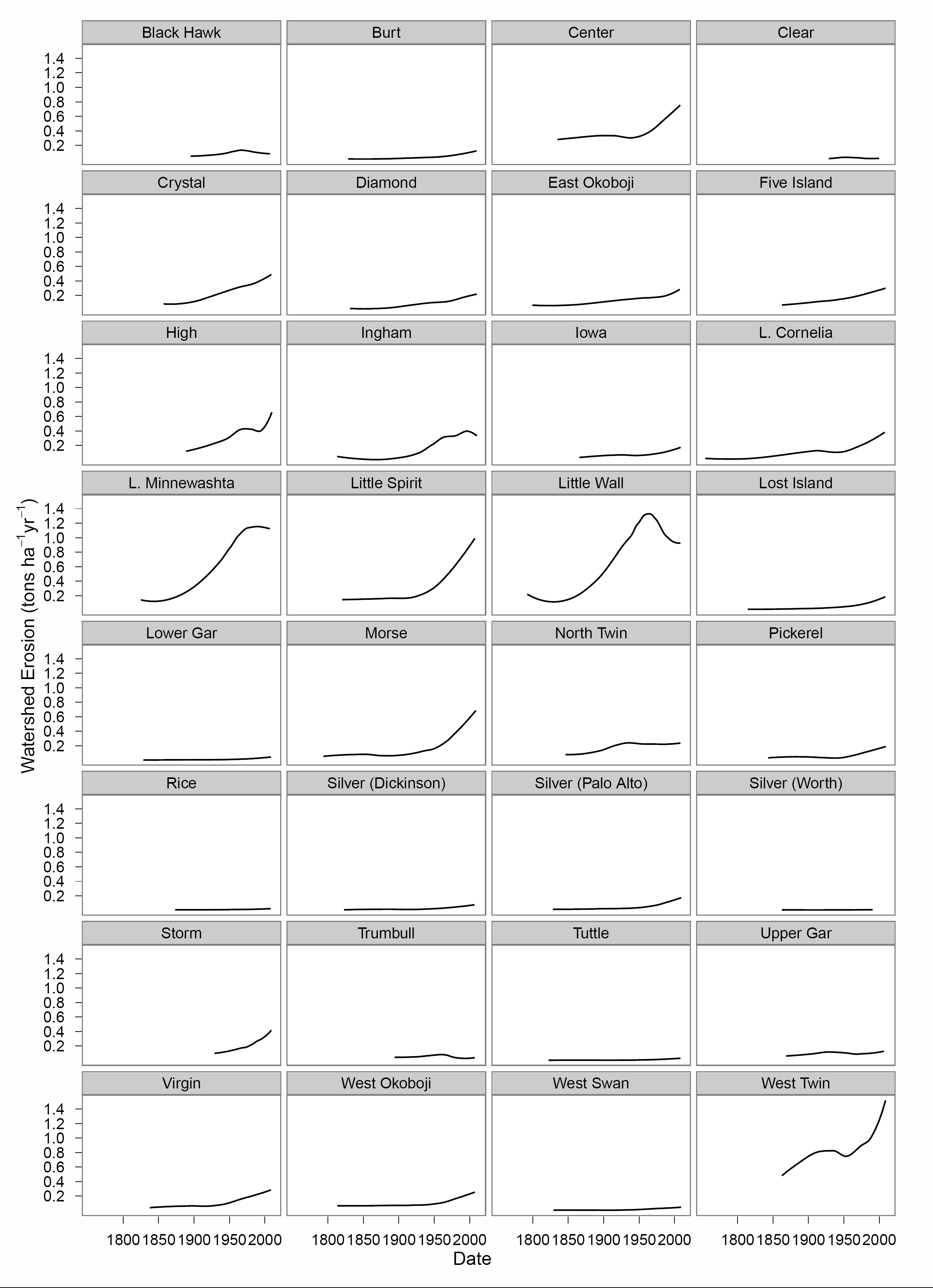

Supplement: Figure S1 — Watershed erosion (tons ha−1 yr−1) versus time for each of the lakes in this study. Black lines represent a LOWESS smoothed fit to the data. (TIF) [file pone.0053554.s001.tif]
