# Supplementary material for: Watershed Sediment Losses to Lakes Accelerating Despite Agricultural Soil Conservation Efforts
Source: PLoS One. 2013 Jan 9;8(1):e53554. doi: 10.1371/journal.pone.0053554 (PMC3541183; doi:10.1371/journal.pone.0053554)
Supplement: Table S1 — Location and water quality characteristics for the 32 lakes in this study. (DOC) [file pone.0053554.s002.doc]

**Table S1.** Location and water quality characteristics for the 32 lakes in this study.

| Lake Name | N UTM Zone 15 | E UTM Zone 15 | Max Depth (m)^†^ | Total P (µg l^-1^)^†^ | Secchi depth (m)^†^ |
| --- | --- | --- | --- | --- | --- |
| Black Hawk Lake | 4685309 | 334050 | 6.1 | 96 | 0.4 |
| Black Hawk Lake (Slough) | 4684938 | 331504 | 0.9 | NA | NA |
| Burt Lake | 4817635 | 388256 | 1.5 | NA | NA |
| Center Lake | 4808658 | 326988 | 4.7 | 90 | 1.0 |
| Clear Lake | 4774952 | 467148 | 5.8 | 47 | 0.8 |
| Crystal Lake | 4786732 | 434818 | 2.4 | 159 | 0.4 |
| Diamond | 4816622 | 322749 | 1.8 | 599 | 0.3 |
| East Lake Okoboji | 4805389 | 328946 | 6.7 | 113 | 1.7 |
| Five Island Lake | 4779873 | 366244 | 6.1 | 122 | 0.5 |
| High Lake | 4795593 | 361639 | 2.4 | 222 | 0.3 |
| Ingham Lake | 4797335 | 362179 | 3.7 | 168 | 0.3 |
| Iowa Lake | 4817559 | 381898 | 2.7 | 138 | 0.3 |
| Lake Cornelia | 4737459 | 443679 | 5.5 | 74 | 0.4 |
| Lake Minnewashta | 4802982 | 327895 | 5.0 | 106 | 1.6 |
| Little Spirit Lake | 4819957 | 328113 | 3.0 | 232 | 0.5 |
| Little Wall Lake | 4723306 | 316567 | 3.7 | 70 | 0.4 |
| Lost Island Lake | 4679752 | 447003 | 4.3 | 76 | 0.5 |
| Lower Gar Lake | 4781524 | 345236 | 1.7 | 100 | 0.7 |
| Morse Lake | 4802028 | 328183 | 1.8 | 217 | 0.5 |
| North Twin Lake | 4743063 | 443514 | 4.0 | 50 | 0.7 |
| Pickerel Lake | 4705866 | 366698 | 1.8 | 304 | 0.2 |
| Rice Lake | 4752202 | 343144 | 4.0 | 533 | 0.1 |
| Silver Lake (Dickinson Co.) | 4804677 | 458470 | 3.4 | 109 | 0.5 |
| Silver Lake (Palo Alto Co.) | 4812521 | 311146 | 2.1 | 153 | 0.3 |
| Silver Lake (Worth Co.) | 4766215 | 346045 | 1.8 | 236 | 0.2 |
| Storm Lake | 4814316 | 466061 | 6.1 | 70 | 0.5 |
| Storm Lake (Inlet) | 4721800 | 319598 | 0.7 | NA | NA |
| Trumbull Lake | 4784188 | 341696 | 1.2 | 234 | 0.2 |
| Tuttle Lake | 4817098 | 370566 | 1.8 | 171 | 0.3 |
| Upper Gar Lake | 4803986 | 328211 | 1.5 | 113 | 1.4 |
| Virgin Lake | 4774045 | 346028 | 1.7 | 361 | 0.2 |
| West Lake Okoboji | 4802764 | 324204 | 41.5 | 22 | 4.2 |
| West Swan Lake | 4801454 | 363840 | 1.6 | 201 | 0.3 |
| West Twin Lake | 4753855 | 440335 | 1.8 | 167 | 0.2 |
| †Data are averages from 2006-2007 Iowa Lake Survey and were collected by the Iowa State | | | | | |
| University Limnology Laboratory (http://limnology.eeob.iastate.edu/lakereport) | | | | | |
